# Supplementary material for: Netcombin: An algorithm for constructing optimal phylogenetic network from rooted triplets
Source: PLoS One. 2020 Sep 18;15(9):e0227842. doi: 10.1371/journal.pone.0227842 (PMC7500971; doi:10.1371/journal.pone.0227842)
Supplement: S4 File — (ZIP) [file pone.0227842.s004.zip › SequenceData-Triplet/sequenceData-10-20-30-0-Triplet/length 500, numseq 40, rate 10^-7/1 seq.pdf]

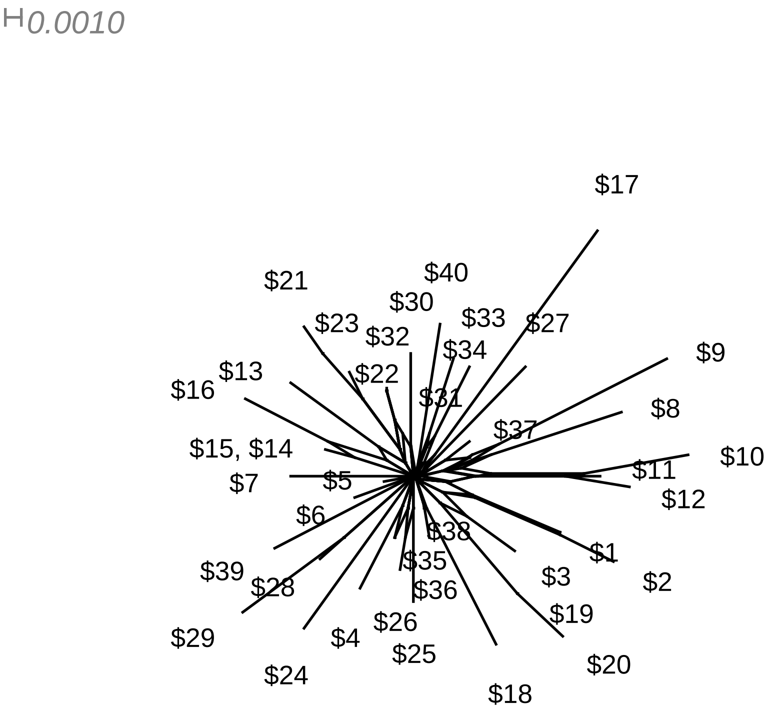
0.0010
$17
$40
$21
$30
$33
$27
$23
$32
$34
$9
$13
$22
$16
$31
$8
$37
$15, $14
$10
$11
$5
$7
$12
$6
$38
$1
$39
$35
$3
$2
$28
$36
$19
$26
$29
$4
$25
$20
$24
$18
